# Identifying All Moiety Conservation Laws in Genome-Scale Metabolic Networks

**DOI:** 10.1371/journal.pone.0100750

**Published:** 2014-07-02

**Authors:** Andrea De Martino, Daniele De Martino, Roberto Mulet, Andrea Pagnani

**Affiliations:** 1 CNR-IPCF, Unità di Roma-Sapienza, Roma, Italy; 2 Dipartimento di Fisica, Sapienza Università di Roma, Roma, Italy; 3 Center for Life Nano Science@Sapienza, Istituto Italiano di Tecnologia, Roma, Italy; 4 Henri-Poincaré-Group of Complex Systems and Department of Theoretical Physics, Physics Faculty, University of Havana, La Habana, Cuba; 5 DISAT and Centre for Computational Sciences, Politecnico di Torino, Torino, Italy; 6 Human Genetics Foundation, Torino, Italy; Université Paris-Sud, France

## Abstract

The stoichiometry of a metabolic network gives rise to a set of conservation laws for the aggregate level of specific pools of metabolites, which, on one hand, pose dynamical constraints that cross-link the variations of metabolite concentrations and, on the other, provide key insight into a cell's metabolic production capabilities. When the conserved quantity identifies with a chemical moiety, extracting all such conservation laws from the stoichiometry amounts to finding all non-negative integer solutions of a linear system, a programming problem known to be NP-hard. We present an efficient strategy to compute the complete set of integer conservation laws of a genome-scale stoichiometric matrix, also providing a certificate for correctness and maximality of the solution. Our method is deployed for the analysis of moiety conservation relationships in two large-scale reconstructions of the metabolism of the bacterium *E. coli*, in six tissue-specific human metabolic networks, and, finally, in the human reactome as a whole, revealing that bacterial metabolism could be evolutionarily designed to cover broader production spectra than human metabolism. Convergence to the full set of moiety conservation laws in each case is achieved in extremely reduced computing times. In addition, we uncover a scaling relation that links the size of the independent pool basis to the number of metabolites, for which we present an analytical explanation.

## Introduction

When studying metabolic networks at the scale of the whole genome, it is often the case that the information required to develop dynamical models is not available, because either kinetic parameters or reaction mechanisms are partially or fully unknown. In many cases, the most reliable information is encoded in the stoichiometry of the reaction network (the stoichiometric matrix) and, partly, in the assignment of reaction reversibility [1]–[4]. Based on these, constraint-based models like Flux Balance Analysis (FBA) have been able to shed light on functional optimality in different contexts, providing (mostly for unicellular organisms) unprecedented predictive power on issues like the organization of reaction fluxes, response to knock-outs, or gene essentiality [5]–[8]. FBA can now almost routinely be performed on genome-scale networks [9]. Stoichiometric matrices however harbor a host of additional physical, biological and functional information [5], [10]–[12], including regulatory (see e.g. extreme pathways [13], [14] and flux modes [15]), robustness (see e.g. the geometry of the FBA solution space [16]–[18]) and statistical (see e.g. the individual distributions of allowed fluxes and flux-flux correlations [18]–[20]) characterizations. Unluckily, the full solution of the problems just listed on genome-scale networks with thousands of reactions and metabolites presents serious computational challenges, as the algorithms currently available do not scale gently with the system size. This is also the case for the identification of moiety conservation laws that we shall consider here [10], [21], [22].

Conservation relationships for concentration variables emerge in biochemical reaction networks from the sheer structure of their input-output stoichiometry [10]. In particular, given a stoichiometric matrix, it is generically possible to find linear combinations of metabolite levels that are due to be constants of motion of the dynamical system governing the time evolution of concentrations and reaction rates. For instance, if the subset of metabolic reactions describing energy metabolism is considered, ATP and ADP would be coupled in every reaction – one is a product whenever the other is a substrate –, so that the aggregate concentration of ATP and ADP would remain constant over time while individual levels may fluctuate in a correlated way. The existence of such relationships has profound consequences. In first place, any intervention aimed at altering the level of a certain metabolite should consider whether its variations are limited or not by conservation laws. Secondly, conservation laws constrain a network's production capabilities, as there clearly cannot be a net stationary output of a compound belonging to a conserved quantity. Therefore, mapping out these conservation laws amounts to obtaining a genome-scale picture of what a cell can (in principle) excrete or make available to processes outside metabolism, in particular secondary metabolism, e.g. the production of pigments and antibiotics. Finally, conserved quantities imply an effective reduction of the number of independent flux or concentration variables in a reaction network, an important aspect especially for dynamical modeling.

The problem of finding generic conservation laws is relatively straightforward to solve with the tools of linear algebra, since, as said above, they correspond to specific (linear) dependencies of the rows of the stoichiometric matrix. When one is interested in the conservation of particular chemical moieties, however, the problem takes a more challenging twist. Indeed, because of intrinsic discreteness, the combinations that describe moiety conservation should only be constructed with non-negative integers. We refer to these particular combinations as ‘moiety conservation laws’ (MCLs), and we shall be interested in finding a basis for MCLs (i.e. a maximal linearly independent set of MCLs) in large, genome-scale metabolic networks. Computing an MCL basis in a large system is much harder than finding linear row-dependencies, as one passes from a linear to an integer-linear programming problem with the concomitant increase of computational complexity up to NP-hard [23], [24]. On the other hand, knowledge of an MCL basis allows, as we shall see, to map out exhaustively a large class of conservation laws in biochemical networks.

The literature on conservation laws in reaction networks, mostly focused on characterizing the so-called semi-positive conservation laws (SPCLs, given by linear combinations of MCL basis vectors), is quite rich. Pioneering mathematical analyses of SPCLs in biochemical systems date back to the early 1990's [21], [25]. Besides clarifying the origin and significance of these invariants, such studies have shown that conservation laws can be encoded in a convex representation of the left kernel of the stoichiometric matrix [25]. Later on [22], a classification of conservation laws has been proposed based on an analogy between properties of the right (extreme pathways) and left null spaces of the stoichiometric matrix. The computational demand of finding MCLs in genome-scale networks has driven further work concerned with the development of *ad hoc* algorithms [26], [27]. After the seminal proposals made in [25], already in [22] a method is presented, that however scales exponentially with the system size and can only be employed for the analysis of rather small networks. The Metabolite Concentration Coupling Analysis (MCCA) and the Minimal Pool Identification (MPI) tools for genome-scale metabolic network analysis were instead introduced in 2005 [28]. The former allows for the identification of subsets of metabolites whose concentrations are coupled within common SPCLs, while MPI helps to determine the SPCLs for individual metabolites. More recently [29], the formal algebraic duality of metabolite producibility and conservation has been exploited to devise a method that relates biomass producibility to nutrient availability, which was then applied to the metabolism of *Escherichia coli*, obtaining a large set of novel putative growth media. Then in [30] a mixed integer-linear programming problem has been posed and solved in order to find the ensemble of all metabolites that appears in SPCLs. Furthermore, in modeling metabolic networks in terms of Petri nets, the problem of finding MCLs has been connected with the search for the so-called P-invariants of the network [31]. It is worth noting that metabolite levels are in principle experimentally accessible (e.g. by mass spectrometry), although such knowledge is mostly used in metabolomic analysis to reconstruct flux patterns.

Despite many efforts, a consistent computational method to determine *all* MCLs in genome-scale networks has remained so far elusive. In this work we construct such a computational method. The technique we propose exploits the above mentioned duality and combines different kinds of algorithms (message passing, Monte Carlo and relaxation). The mathematical background and the structure of the method we employ are discussed in ‘Materials and Methods’. As case studies, we have considered different reconstructions of the metabolic network of the bacterium *E. coli* and six tissue-specific human metabolic networks derived from the Recon-2 database [32]. In particular, we have been able to identify in each case *all* MCLs in different conditions (see ‘Results’). Furthermore, by studying *E. coli* we have uncovered a relation between the number of pools and the size of a network (number of metabolites and/or reactions), a theoretical justification for which is also discussed, before reporting our conclusions.

## Materials and Methods

### Methods

#### Background

Given a metabolic network encoded by the stochiometric matrix 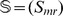, where 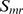 is the stochiometric coefficient of metabolite 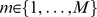 in reaction 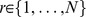 (with the standard sign convention to distinguish substrates from products), the time evolution of the concentration vector 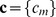 satifies
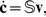
(1)where 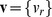 is the vector of reaction fluxes and we have assumed that the stoichiometry of metabolite exchanges with the environment is included in 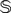. Consider a linear combination 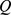 of concentration variables with fixed coefficients 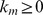, i.e.
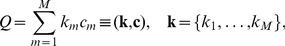
(2)where the bracket 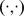 stands for the scalar product. Clearly,
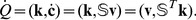
(3)So if 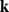 belongs to the left null-space of 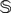, that is if
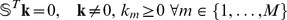
(4)then the aggregate concentration variable 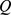 is conserved in *any* flux state 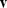. Following [21], we shall generically call a conserved metabolite pool like 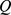, defined by a vector 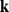 satisfying (4), a ‘semi-positive conservation law’ (SPCL). From a physical viewpoint, a SPCL represents an invariance constraint that is required to be satisfied by trajectories of the dynamics of the system, i.e. by solutions of (1) with given specifications of how 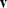 depends on 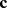. In principle, every vector 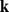 belonging to the left kernel of 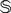 (without sign restriction) defines a conserved quantity, to which we can refer generically as a ‘conservation law’. The total number of linearly independent conservation laws of this type equals the dimension of the left null-space of 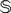, i.e. 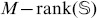. This number also provides an upper bound to the number of linearly independent SPCLs, namely
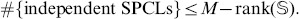
(5)The restriction to 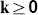 in (4) allows for a more straightforward interpretation of quantities like 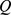. Considering, for instance, the toy network formed by the three reactions
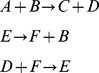
(6)a simple calculation shows that two conserved quantities exist, i.e. 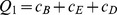 and 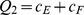. However, 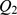 could be also written as 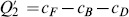. While both 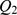 and 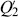 are conserved quantities, 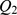 can be interpreted to be a total enzyme mass if 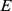 and 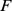 represent, respectively, a bound and a free enzyme species. A similar physical interpretation is harder to imagine for 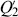 (and the situation rapidly becomes more complicated in larger networks).

Among solutions of (4), those for which the coefficients 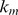 are *non-negative integers* can be fully rationalized in chemical terms as related to the conservation of moieties, groups or chemical elements. We shall hereafter define a ‘moiety conservation law’ (MCL) as a solution of
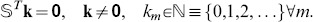
(7)The problem we face here concerns the identification of all MCLs of a given stoichiometric matrix 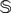. Furthermore, we will be interested in constructing an integer basis of the left null space of 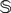 through which all SPCLs can be obtained as linear combinations with real coefficients. From a mathematical view point, finding such a basis represents a variation of the more general problem of computing the minimal integer Hilbert basis of the polyhedral cone defined by the left null-space of the stoichiometric matrix, which is known to be NP-hard [33], [34]. Although some exact deterministic algorithms are available [35], [36], their computational costs become too high when the underlying network is sufficiently large. In particular, for the sizes relevant to genome-scale metabolic modeling (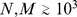) one always runs into the combinatorial explosion of computation times.

Incidentally, if this integer basis suffices to generate all conservation laws via linear combinations with real coefficients, then all conservation relationships encoded in 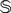 are SPCLs and the number of MCLs in the basis saturates the bound (5). Otherwise, 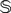 necessarily allows for at least one conservation law that cannot be expressed through a SPCL but also involves negative coefficients. In such cases, we have 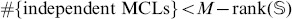 and an integer basis of the left kernel, rigorously speaking, does not exist.

It is very important to stress that a high computational complexity does not necessarily imply the existence of an exponential number of elements of the MCL basis (as a matter of fact, we shall see that the basis size grows roughly linearly with the network size); rather, it quantifies the time required to find a single element of the basis in the worst case. Roughly speaking, combinatorial explosion of computing times occurs when one has to go through an exponential number of candidates (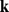-vectors in our case) before finding a solution of (7). It is rather intuitive that finding all solutions of (7) by a deterministic procedure would require exploring the entire space of 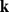-vectors, which assuming non-zero upper bounds for the 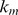
's is indeed exponentially large in the number of metabolites.

In order to find all independent solutions to (7), stochastic strategies are therefore mandatory. In brief, we shall map this task to a global optimization problem whose solution can be retrieved via stochastic algorithms known to be exact in special situations, a kind of approach that has been used before with considerable success in the solution of other NP-complete and NP-hard problems [18], [37]–[39]. Our strategy is divided in three steps: (a) compute a list of all metabolites belonging to at least one MCL; (b) construct an integer basis for MCLs from that list; (c) check that 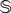 does not allow for any further MCL. Step (a) can be carried out in different ways, starting, as we shall see, from a straightforward analysis of the kernel of 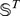. We shall also discuss here a more involved but more informative approach based on a message-passing procedure. Step (b) will be done by Monte Carlo and step (c) by a relaxation algorithm. The conceptual implementation of each step is sketched below and a C++ code performing the complete procedure can be downloaded from http://chimera.roma1.infn.it/SYSBIO/, together with a test case (the E. coli iAF1260 network). Further details and work flow are given in the Text S1.

#### Step (a): finding all metabolites belonging to at least one MCL

A first, elementary preprocessing step may consist in computing the left kernel of 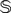, e.g. by Gaussian elimination. Evidently, every vector in a basis of 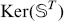 is a generic, linearly independent conservation law, i.e. a non-null solution of 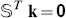 with real-valued 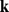. Eventually, some of these vectors will be such that 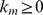 for each 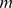, i.e. will be outright SPCLs. The search for MCLs can be carried out among metabolites that appear in this basis with positive components. Furthermore, the size of such a basis is an upper bound for the maximum number of linearly independent MCLs. In order to compute the left kernel, we resort to a Gaussian elimination method with partial scaled pivoting, a technique currently employed to deal effectively with possible ill-conditioning of the stoichiometric matrix under control. We however cross-checked results, in every case, with the results obtained by the robust routines employed by both *Mathematica* and *MatLab* softwares. Ultimately, though, the relaxation algorithm discussed below provides a definitive certificate for correctness, in the sense that convergence only occurs when all kernel vectors have been found (and removed from the stoichiometric matrix, see below).

A more complex but (in our view) more rewarding alternative is suggested by the following considerations. Because MCLs are represented by solutions of (7), one could obtain a statistical picture of the set of MCLs by computing the marginals of the probability distribution
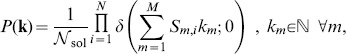
(8)where 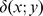 is the Kronecker delta function (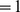 if 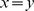 and 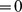 otherwise) and 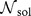 stands for the number of solutions of (7). Indeed, each marginal
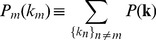
(9)represents the probability that the 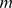-th component of the 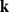 vector attains a value 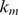 over the solution space of (7). Disposing of all such marginals is equivalent to disposing of the list of metabolites belonging to at least one MCL, since 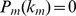 implies that metabolite 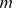 does not belong to any MCL. Actually, the set of marginals provides much more information than that: in fact, by definition,
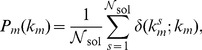
(10)where in the last equivalence we stressed the probabilistic interpretation of the marginal as the histogram of the 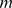-th coordinate over the solutions 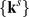, 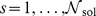. So, 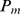 is proportional to the number of distinct MCLs to which metabolite 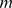 belongs.

Unluckily, a direct evaluation of (10) requires computing a sum over 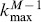 terms (assuming 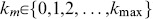), which becomes computationally infeasible for 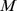 larger than a few tens. We can however estimate each marginal effectively by resorting to message-passing (MP) techniques. MP is an efficient computational strategy (with a running time scaling linearly, as opposed to exponentially, with the number of variables) that is formally exact on locally tree-like networks [40]–[42] and is extensively used as a heuristic procedure to solve hard computational problems defined on sparse or even loopy networks [37], [43], [44]. It has been also applied previously to the analysis of metabolic networks [18].

In short, we have devised a MP algorithm to compute the marginals (10) and obtain a full statistical representation of the space of MCLs. Details are given in the Text S1. Upon convergence, when all marginal probability distributions are evaluated, one disposes of a list of metabolites belonging to at least one MCL (with the additional information described above). The following step concerns the construction of an integer basis for MCLs from this list.

#### Step (b): constructing the basis

Because MP will have considerably pruned the set of metabolites (thereby greatly reducing the number of variables: we shall now denote by 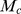 the number of metabolites belonging to at least one MCL), the most convenient method to explore the structure of individual MCLs is Monte Carlo. Indeed, the problem of finding the integer solutions of (7) can be mapped onto that of finding the minima of the fictitious, discrete ‘energy function’ given by
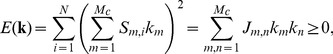
(11)where 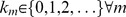 and
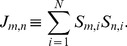
(12)Note that 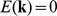 if 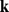 satisfies (7). Therefore MCLs correspond to the minima of 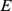. Several controlled and optimized Monte Carlo methods are available to compute the minima of functions like (11) [45]. More specifically, these protocols (the simplest of which is probably the Metropolis scheme) are capable of sampling vectors 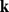 distributed according to
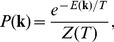
(13)where 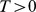 is a parameter and 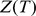 a normalization factor. The minima are recovered upon slowly decreasing 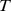 (‘annealing’) in the limit 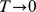.

In this work, in order to retrieve the individual MCLs as ground states of 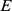, we have performed iterated Metropolis-based annealings to minimize the energy (11) as detailed in the Text S1. Eventual linear dependencies among the retrieved minima can be resolved by Gaussian elimination to yield an actual non-negative integer basis for the space of MCLs. In case superpositions of independent MCLs (for which 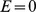 as well) are identified as minima of 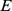 by Monte Carlo, they can be easily reduced by iteratively subtracting the other independent MCLs found, taking care to maintain the non-negativity constraint for the coefficients 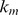.

#### Step (c): checking completeness

As in [29], we can exploit the connection between (7) and its dual system [21], i.e.
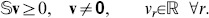
(14)The solution spaces of (7) and (14) are linked by the Motzkin theorem of the alternative, which can be stated as follows:

**Theorem** (Motzkin, 1936). *Consider any arbitrary subset R of rows of* 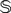. *Then, either there exists a solution* **v** ^*^ *to system (14) such that all inequalities corresponding to the subset R hold strictly, or system (7) has a solution* **k** ^*^, *with* 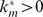 *for each* 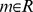.

In essence, Motzkin's result guarantees that solutions of (14) verify strict *equalities* for metabolites belonging to MCLs. This is rather intuitive if one interprets strict inequalities in (14) as conditions for metabolite producibility [29], [46]. Luckily, a solution of a subset of constraints in (14) with strict *inequalities* can be found very efficiently by relaxation algorithms (e.g. MinOver [47] or Motzkin's scheme [48]). These classic methods work by correcting iteratively (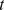 being the step) the least unsatisfied constraint, according to the scheme
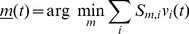
(15)

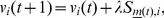
(16)

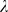
 being a parameter that can be fixed in different ways, from a constant (as in MinOver [49]) to a quantity proportional to the amount by which the constraint is violated (as in the so-called Motzkin scheme [48]). The above dynamics converges to a solution, if one exists, in polynomial time.

Therefore a simple numerical check to confirm that all MCLs have been found consists in looking for a solution of (14) with strict inequalities for all 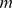
's remaining after having removed from 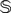 the rows corresponding to metabolites belonging to at least one MCL: when all MCLs have been found, a solution necessarily exists and relaxation converges to it. Further detailes are reported in the Text S1. This completeness analysis finalizes our protocol.

### Materials

We shall apply the method described above to find MCL bases for two reconstructions of *E. coli*'s metabolic networks of rather different sizes, namely iJR904 [4], with 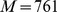 chemical species and 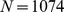 reactions, and iAF1260 [50], with 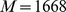 and 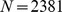. These numbers refer to the sizes of the respective stoichiometric matrices including all uptakes but excluding the biomass reaction. Two limiting cases for the choice of the exchange fluxes will be considered. First, we shall analyze MCLs formed in a ‘rich medium’, where all uptake reactions are active. Then, we shall look at the case of ‘minimal medium’, by studying MCLs after having eliminated part of the intakes, while keeping exchange reactions for ca2, fe2, glc-D, h2o, h, k, mg2, mn2, na1, nh4, o2, pi, so4 and zn2. (In the latter case a much larger number of MCLs is to be expected.) We shall see that, while for iAF1260 independent MCLs suffice to generate all conservation laws as linear combinations with non-negative coefficients (in other words, the bound (5) is saturated), the model iJR904 presents one conserved quantity that cannot be expressed as a linear superposition of MCLs with non-negative coefficients.

We have furthermore computed the independent MCLs emerging in 6 tissue-specific reconstructions of human metabolic networks derived from the reactome Recon-2 [32] (bone marrow, breast glandular, heart muscle, hippocampus glial and neuronal and liver hepatocytes), as well as in the entire reactome. The latter case serves mainly to obtain computing times in a worst case (largest reconstruction tested). MCLs for the human networks were obtained using stoichiometric matrices that include all uptakes and exclude the (eventual) biomass reaction.

## Results

### E. coli iAF1260

Table 1 lists the independent MCLs found for iAF1260 with a ‘rich medium’. They are 38 in total, matching exactly the dimension of the left kernel of the stoichiometric matrix.

**Table 1:** The 38 independent MCLs found for the network iAF1260.

MCL ID	Size	Conserved species	Formula
1–19	2	tRNA	alatrna[c] + trnaala[c], argtrna[c] + trnaarg[c], asntrna[c] + trnaasn[c], asptrna[c] + trnaasp[c], cystrna[c] + trnacys[c], glntrna[c] + trnagln[c], glutrna[c] + trnaglu[c], glytrna[c] + trnagly[c], histrna[c] + trnahis[c], iletrna[c] + trnaile[c], leutrna[c] + trnaleu[c], lystrna[c] + trnalys[c], phetrna[c] + trnaphe[c], protrna[c] + trnapro[c], sertrna[c] + trnaser[c], thrtrna[c] + trnathr[c], trptrnatrp[c] + trnatrp[c], tyrtrnatyr[c] + trnatyr[c], valtrnaval[c] + trnaval[c]
20–23	2	missing transport and leaves	arbt6p[c] + hqn[c], cyan[c] + tcynt[c], dms[c] + dmso[c], tma[c] + tmao[c]
24–28	2	lipoprotein	alpp[p] + lpp[p], dsbaox[p] + dsbard[p], dsbcox[p] + dsbcrd[p], dsbdox[c] + dsbdrd[c], dsbgox[p] + dsbgrd[p]
29–31	2	redox enzymes	fldox[c] + fldrd[c], grdox[c] + grxrd[c], trdox[c] + trdrd[c]
32	3	tRNA	fmettrna[c] + mettrna[c] + trnamet[c]
33–34	3	selenium compounds	sectrna[c] + seln[c] + selnp[c], sectrna[c] + sertrnasec[c] + trnasecys[c]
35	3	biotin	btn[c] + btnso[c] + s[c]
36	3		8aonn[c] + amob[c] + pmcoa[c]
37	6		8aonn[c] + btn[c] + btnso[c] + dann[c] + dtbt[c] + pmcoa[c]
38	53	ACP	3haACP[c] + 3hcddec5eACP[c] + 3hcmrs7eACP[c] + 3hcpalm9eACP[c] + 3hcvac11eACP[c] + 3hddecACP[c] + 3hdecACP[c] + 3hhexACP[c] + 3hmrsACP[c] + 3hoctACP[c] + 3hoctaACP[c] + 3hpalmACP[c] + 3ocddec5eACP[c] + 3ocmrs7eACP[c] + 3ocpalm9eACP[c] + 3ocvac11eACP[c] + 3oddecACP[c] + 3odecACP[c] + 3ohexACP[c] + 3omrsACP[c] + 3ooctACP[c] + 3ooctdACP[c] + 3opalmACP[c] + ACP[c] + acACP[c] + actACP[c] + apoACP[c] + but2eACP[c] + butACP[c] + cddec5eACP[c] + cdec3eACP[c] + dcaACP[c] + ddcaACP[c] + hdeACP[c] + hexACP[c] + malACP[c] + myrsACP[c] + ocACP[c] + ocdcaACP[c] + octeACP[c] + palmACP[c] + t3c11vaceACP[c] + t3c5ddeceACP[c] + t3c7mrseACP[c] + t3c9palmeACP[c] + tddec2eACP[c] + tdeACP[c] + tdec2eACP[c] + thex2eACP[c] + tmrs2eACP[c] + toct2eACP[c] + toctd2eACP[c] + tpalm2eACP[c]

The suffixes [c] and [p] indicate the presence of that species in the cytoplasm and periplasm, respectively, in agreement with the compartmentation indicated in the reconstruction.


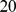
 of them (numbers 1–19 and 32) are formed by a tRNA in two forms: free and bound to its corresponding amino-acid. To have a physical interpretation, we note that if a model possesses a conserved quantity corresponding to a chemical moiety, then that model is closed with respect to that moiety, in the sense that it does not allow for changes in the level of that particular chemical group. In this sense, MCLs based on a tRNA reflect the fact that, in the model where they have been found, the expression of each tRNA is necessarily constant (more precisely, it is assumed to change on time scales longer than those over which metabolite levels equilibrate).

Compounds in MCL 20, arbutin 6-phosphate (arbt6p) and hydroquinone (hqn) only occur in one reaction (arbutin 6-phosphate glucohydrolase: arbt6p + h2o → g6p + hqn). From a topological point of view, they represent ‘leaves’ of the reaction network, in the sense that arbt6p is not produced by any reaction while hqn is not consumed by any reaction. The fact that their overall level is invariant is merely due to this peculiar geometric property.

MCL 21 is composed by hydrogen cyanide (cyan) and thiocyanate (tcynt) in their cytoplasmic form. Interestingly, the aggregate concentration of these compounds is conserved despite the fact that, in the rich medium, there are uptakes for both. This is due to the fact that the model lacks reactions that transport the periplasmic species into the cytoplasm. Exactly the same situation holds for the MCLs 22 and 23, formed by dymethyl-sulfide (dms) and -sulfoxide (dmso), and by thrymethylamine (tma) and thrymethylamine-N-oxide (tmao), respectively.

MCLs 24–28 express the conservation of the level of lipoproteins (apolipoprotein, disulfide isomerase I and II, disulfide interchange and oxidase). Notice that MCL 28 is the only one to involve periplasmic species exclusively.

MCLs 29–31 describe the invariance of the level of the redox enzymes flavodoxin (fldox), glutaredoxin (grdox) and thioredoxin (trdox).

Compounds in MCLs 33 and 34 are all based on the element selenium, with respect to which the model is closed (i.e. there are no uptakes of selenium-based compounds). We also note from this example that independent MCLs can be overlapping, in the sense that the same compound (sectrna in this case) may belong to different conserved quantities. In such cases, the levels of metabolites in the overlapping groups become effectively coupled, allowing to re-cast the moiety conservation laws associated to the overlapping pools in simpler terms. In the present case, for instance, the fact that sectrna is shared by MCLs 33 and 34 corresponds to the conservation of the single quantity 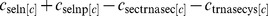.

MCL 35 reflects the conservation of the level of biotin, while the sulfur atom is a leaf of the network, appearing only in the biotin synthase reaction.

MCL 36 expresses the invariance of the aggregate level of 8-amino-7-oxononanoate (8aonn), S-adenosyl-4-methylthio-2-oxobutanoate (amob) and pimeloyl-coa (pmcoa), while MCL 37 involves 8aonn, pmcoa, the biotin compounds of pool 35 (btn, btnso), plus 7,8-diaminononanoate (dann) and dethiobiotin (dtbt), providing a further instance of overlapping independent pools. At odds with previously described MCLs, these appear lack a clear interpretation in terms of a chemical moiety.

Finally, MCL 38 represents the conservation of the level of acyl carrying protein (ACP).

We can argue that a suitable set of additional uptakes (comprising tRNAs, selenium, disulfide proteins, the aforementioned redox enzymes, biotin, and ACP), together with the missing periplasm-cytosol transport reactions (for cyan, tma and dms), will render the iAF1260 network completely open, thereby allowing for the possibility that the levels of each of the chemical moieties appearing therein are altered. Eliminating uptakes, on the other hand, will generate additional MCLs. Table 2 reports the 36 additional independent MCLs that occur in iAF1260 in a ‘minimal medium’ containing only ca2, fe2, glc-D, h2o, h, k, mg2, mn2, na1, nh4, o2, pi, so4 and zn2 (i.e. by allowing for 14 of the 299 possible uptakes). A close inspection reveals that many of these MCLs emerge from the lack of uptakes for elements like silver (39), cadmium (40), nickel (41), molybdenum (42), cobalt (43), tungsten (44), mercury (46), chloride(47), arsenic (55) and copper (64). A more detailed biochemical analysis is required to interpret the remaining MCLs.

**Table 2:** The 36 additional independent MCLs that are found in iAF1260 in a ‘minimal medium’.

MCL ID	Size	Formula
39	2	ag[c] + ag[e]
40–47	3	cd2[c] + cd2[e] + cd2[p], ni2[c] + ni2[e] + ni2[p], mobd[c] + mobd[e] + mobd[p], cobalt2[c] + cobalt2[e] + cobalt2[p], tungs[c] + tungs[e] + tungs[p], met-D[c] + met-D[e] + met-D[p], hg2[c] + hg2[e] + hg2[p], cl[c] + cl[e] + cl[p]
48–57	4	betald[c] + glyb[c] + glyb[e] + glyb[p], bbtcoa[c] + gbbtn[c] + gbbtn[e] + gbbtn[p], 4hoxpacd[e] + 4hoxpacd[p] + tym[e] + tym[p], dms[e] + dms[p] + dmso[e] + dmso[p], cyan[e] + cyan[p] + so3[e] + so3[p], 3sala[c] + so2[c] + so2[e] + so2[p], gdp[e] + gdp[p] + gtp[e] + gtp[p], aso3[c] + aso3[e] + aso3[p] + aso4[c], 34dhpac[e] + 34dhpac[p] + dopa[e] + dopa[p], tma[e] + tma[p] + tmao[e] + tmao[p]
58	6	feoxam-un[c] + feoxam-un[e] + feoxam-un[p] + feoxam[c] + feoxam[e] + feoxam[p]
59	6	cpgn-un[c] + cpgn-un[e] + cpgn-un[p] + cpgn[c] + cpgn[e] + cpgn[p]
60	6	fecrm-un[c] + fecrm-un[e] + fecrm-un[p] + fecrm[c] + fecrm[e] + fecrm[p]
61	6	fe3hox-un[c] + fe3hox-un[e] + fe3hox-un[p] + fe3hox[c] + fe3hox[e] + fe3hox[p]
62	6	arbtn-fe3[c] + arbtn-fe3[e] + arbtn-fe3[p] + arbtn[c] + arbtn[e] + arbtn[p]
63	6	acgal1p[e] + acgal1p[p] + acgal[e] + acgal[p] + udpacgal[e] + udpacgal[p]
64	6	cu2[c] + cu2[e] + cu2[p] + cu[c] + cu[e] + cu[p]
65	6	cyan[e] + cyan[p] + tcynt[e] + tcynt[p]
66	6	chol[c] + chol[e] + chol[p] + g3pc[c] + g3pc[e] + g3pc[p]
67	7	mercppyr[c] + tcynt[c] + tcynt[e] + tcynt[p] + tsul[c] + tsul[e] + tsul[p]
68	7	pac[c] + pacald[c] + pacald[e] + pacald[p] + peamn[e] + peamn[p] + phaccoa[c]
69	9	g3pi[c] + g3pi[e] + g3pi[p] + inost[c] + inost[e] + inost[p] + mi1p-D[c] + minohp[e] + minohp[p]
70	9	5prdmbz[c] + adocbl[c] + adocbl[e] + adocbl[p] + cbl1[c] + cbl1[e] + cbl1[p] + dmbzid[c] + rdmbzi[c]
71	10	crnDcoa[c] + crn-D[c] + crn-D[p] + crn[c] + crn[e] + crn[p] + crncoa[c] + ctbt[c] + ctbt[p] + ctbtcoa[c]
72	10	(2) dopa[e] + (2) dopa[p] + (2) h2o2[e] + (2) h2o2[p] + o2s[e] + o2s[p] + (2) peamn[e] + (2) peamn[p] + (2) tym[e] + (2) tym[p]
73	11	aragund[c] + garagund[c] + gfgaragund[c] + (2) o16a2und[p] + (3) o16a3und[p] + (4) o16a4colipa[e] + (4) o16a4colipa[p] + (4) o16a4und[p] + o16aund[c] + o16aund[p] + ragund[c]
74	12	adocbi[c] + adocbip[c] + adocbl[c] + adocbl[e] + adocbl[p] + agdpcbi[c] + cbi[c] + cbi[e] + cbi[p] + cbl1[c] + cbl1[e] + cbl1[p]

Numbers in parenthesis refer to the values of *k_m_* for the specific metabolites, when different from 1.

Notice that, while 72 of the MCLs discussed above correspond to solutions of (7) with 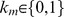 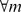, MCL 72 has 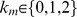 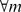 while MCL 73 has 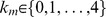 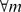. This shows that, while in general identifying SPCLs cannot be treated as a Boolean problem, the range of values of 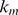 to be considered in (7) can be relatively small.

### E. coli iJR904

For sakes of comparison, in Tables 3 and 4 we report the independent MCLs found in the iJR904 reconstruction of *E. coli*'s metabolism in the ‘rich’ (all uptakes allowed) and ‘minimal’ (defined in the same way as for iAF1260) media, respectively. One can see that, in essence, the MCLs of iJR904 are included among those of iAF1260, with some simplifications. For instance, the pool related to ACP conservation (number 17 in Table 3) is smaller in iJR904 than it is in iAF1260. Notice also that MCL 29 in Table 4 displays two anomalous coefficients 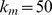, due to the effective, non-integer stoichiometry with which the corresponding compounds occur in the reconstruction. When a compound is represented by a small non-integer coefficient in a reaction, solutions of (7) will typically take on large values of 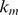.

**Table 3:** The 17 independent MCLs found for the complete network iJR904.

MCL ID	Size	Formula
1–10	2	trdrd[c] + trdox[c], seln[c] + selnp[c], trnaglu[c] + glutrna[c], dms[c] + dmso[c], tmao[c] + tma[c], hqn[c] + arbt6p[c], tcynt[c] + cyan[c], 3dhguln[c] + 23doguln[c], idp[c] + itp[c], acon_T[c] + aconm[c]
11–14	3	ctbt[c] + gbbtn[c] + crn[c], g3pi[c] + inost[c] + mi1p_D[c], 8aonn[c] + amob[c] + pmcoa[c], bbtcoa[c] + crncoa[c] + ctbtcoa[c]
15	4	pacald[c] + peamn[c] + pac[c] + phaccoa[c]
16	6	pmcoa[c] + 8aonn[c] + dann[c] + dtbt[c] + btn[c] + btnso[c]
17	12	apoACP[c] + acACP[c] + actACP[c] + ACP[c] + malACP[c] + ddcaACP[c] + octeACP[c] + myrsACP[c] + palmACP[c] + hdeACP[c] + tdeACP[c] + 3hmrsACP[c]

The suffix [c] indicates that the compound occurs in the cytoplasm.

**Table 4:** The 14 additional independent MCLs that are found in iJR904 in a “minimal medium”.

Pool ID	Size	Formula
18–23	2	fuc1p_L[c] + fuc1p_L[e], dmso[e] + dms[e], nad[e] + amp[e], met_D[e] + met_D[c], tmao[e] + tma[e], gbbtn[e] + crn[e]
24–27	3	glyb[c] + betald[c] + glyb[e], taur[e] + taur[c] + aacald[c], gbbtn[c] + gbbtn[e] + bbtcoa[c], tsul[e] + tsul[c] + tcynt[c]
28	5	ctbtcoa[c] + ctbt[c] + crncoa[c] + crn[c] + crn[e]
29	5	g3pc[c] + chol[c] + (50) pc_EC[c] + (50) agpc_EC[c] + chol[e]
30	6	rdmbzi[c] + adocbl[c] + cbl1[c] + cbl1[e] + 5prdmbz[c] + dmbzid[c]
31	7	adocbip[c] + agdpcbi[c] + adocbl[c] + cbl1[c] + cbl1[e] + adocbi[c] + cbi[c]

Numbers in parenthesis refer to the values of *k_m_* for the specific metabolites, when different from 1.

Considering the ‘rich medium’, it is interesting to note that, even though Table 3 exhausts all of its MCLs (17 in total), the existence of an additional conservation law is revealed by studying the left kernel of the stoichiometric matrix, whose dimension turns out to be 18 rather than 17. The corresponding conserved quantity cannot be expressed as a linear combination of MCLs with non-negative coefficients. In particular, it is formed by the levels of 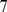 metabolites, with the formula
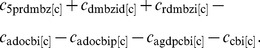
(17)Once we move to the ‘minimal medium’, however, the chemical species pertaining to this conserved quantity fall into well defined MCLs, namely numbers 30 and 31 in Table 4. The additional 14 independent MCLs generated by iJR904 in a ‘minimal medium’ suffice to describe all conservation laws for this network.

### Human metabolic network reconstructions

Finally we turn our attention to the independent MCLs arising within the human reactome Recon-2 and in six of the tissue-specific networks derived from it [32]. The full details of the MCLs we found are provided as Material S1. To summarize results, the sizes of the bases (number of independent MCLs) and the convergence times of the method (specifically, of the C++ code downloadable from http://chimera.roma1.infn.it/SYSBIO/) are reported in Table 5.

**Table 5:** Summary of the number of independent MCLs, of the overall number of metabolites included in at least one MCL basis element and of the convergence times of our algorithm for the networks being examined.

Network	N	M	# MCLs	# metabolites in MCLs	convergence time (s)
E. Coli core	94	72	5	12	<10^−6^
E. Coli iJR904	1074	761	17	52	5.47
E. Coli iAF1260	2381	1668	38	131	2.86
Bone_Marrow	2274	1579	85	294	78
Breast_glandular	2484	1716	88	279	1.7
Heart_muscle	2692	1929	104	346	2.1
Hippocampus_glial	1576	1033	107	414	49.21
Hippocampus_neuronal	2303	1588	94	275	1.47
Liver_hepathocytes	3040	2166	95	309	2.89
Recon 2	7440	5063	397	1135	216

Convergence times were measured for the C++ code thaht can be downloadad from http://chimera.roma1.infn.it/SYSBIO/ on an Intel Dual Core running at 3.06 GHz. 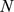 and 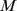 denote, respectively, the numbers of reactions and metabolites that characterize the reconstructions.

We note that if a MCL occurs in Recon-2 and its metabolites are present in a specific subnetwork, then the same MCL will be present in the subnetwork as well. An example is given by the aggregate level of mithocondrial NAD and NADPH, which is conserved in the complete reactome and in all subnetworks we tested. On the other hand, a MCL may be present in a tissue specific network but not in the full reactome, as is the case for the MCL describing the conservation of the sodium cathione, which occurs in all subnetworks we tested but not in Recon-2, due to the presence of a sodium uptake in the latter but not in the tissue-specific reconstructions.

Only one of the networks we analyzed presents conservation laws that cannot be derived from MCLs, namely the bone marrow cell metabolic network. In this particular case, the convergence time of the method suffers from the lack of a stopping criterion in terms of the dimension of the kernel, so that verification of completeness by a relaxation dynamics is mandatory. In just two other cases convergence times exceed a few seconds, namely for the entire reactome (whose size is over twice as large as that of any other network we tested) and the hippocampus glial network. In the latter case, a large MCL related to the conservation of coenzyme A is present, whose discovery requires a longer computing time. In the worst case (Recon-2), however, the time for the full procedure to converge and output the basis vectors is just over 3 minutes on an Intel Dual Core at 3.06 GHz.

Finally, we note that the number of MCLs and of metabolites involved therein considerably exceed those found in the *E. coli* reconstructions, even though the network sizes are comparable at least with iAF1260. Considering that a reduced structure of MCLs correlates with larger production capabilities, this suggests that bacterial metabolic networks may be intrinsically designed by evolution to cover a broader spectrum of production profiles than their human counterparts. It will be important to validate this scenario on future, more accurate reconstructions of human metabolism.

### Scaling of the number of independent MCLs with the network size

**Figure 1:**
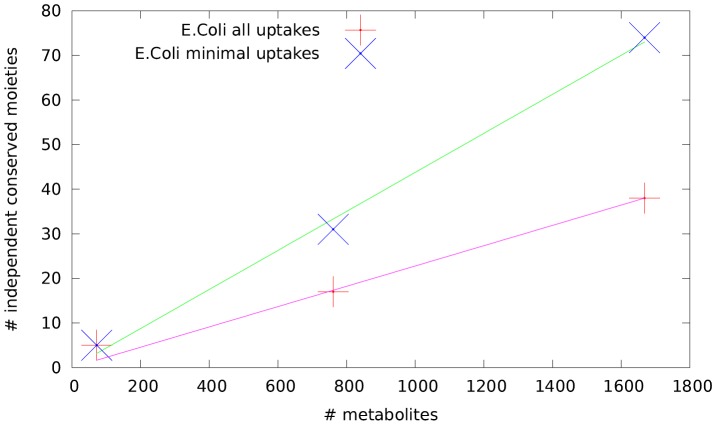
Scaling of the number of independent MCLs with the network size. Size of the MCL basis as a function of the number of metabolites (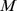) for three *E. coli* metabolic network reconstructions in ‘rich’ (all uptakes active) and ‘minimal’ media.

In Figure 1 we display the size of the MCL basis (i.e. the number of independent MCLs) as a function of the network size (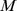 in this plot) for the E. coli networks we have considered above as well as for a smaller reconstruction, namely the core matrix of the iAF1260 model [50] (formed by 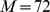 metabolites that interact through 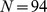 reactions), both for the ‘rich’ and ‘minimal’ media. The ‘rich medium’ for the *E. coli* core network allows for 20 uptakes, 5 of which (glc-D, o2, nh4, pi, h2o) survive in the ‘minimal medium’. This network is easily seen to present, in both media, 5 MCLs with straightforward biochemical meaning: nad 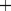 nadh (nad conservation), nadp 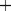 nadph (nadp conservation), q8 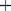 q8h2 (coenzyme-Q conservation), amp 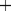 arp 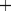 atp (adenylate moiety conservation) and coa 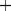 accoa 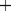 succoa (coenzyme-A conservation). It appears that the number of independent MCLs scales approximately linearly with the network size. (A similar study for the human tissue-specific networks is less fruitful, as the network sizes are similar in those cases and indeed the number of independent MCLs retrieved is roughly constant, as shown in the Text S1.) While the investigation of a larger family of networks is needed to characterize this regularity more thoroughly, it is instructive to analyze the origin of this scaling behaviour. Interestingly, some insight can be obtained from the analysis of random networks.

Consider an ensemble of ‘random metabolic networks’ with 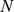 reactions and 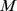 compounds, such that each stoichiometric coefficient is chosen randomly and independently with probabilities (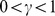)
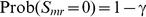
(18)

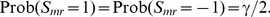
(19)The parameter 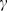 rules the average connectivity of the network. In particular, we will assume that 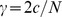 (with 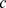 a constant), and consider the limit 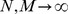, keeping a fixed ratio 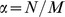. In this limit, the above model generates sparse stoichiometric matrices with Poisson distribution for the in- and out-degrees, with average values 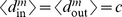 for metabolites and 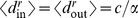 for reactions, respectively. (In real networks, the degree distribution of metabolites is known to have heavy tails due to the presence of ubiquitous compounds like water, ATP, etc. whose connectivities typically grow with the network size. On the other hand, the degree distribution of the remaining metabolites follows a Poissonian law to a good approximation.) By definition, the overall number of MCLs of size 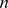 (i.e. involving 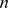 chemical species) is given by
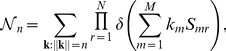
(20)where 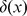 denotes Dirac's 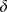-function, we assume 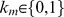 for sakes of simplicity for each 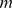, and 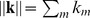. 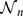 as defined above however includes all linear combinations of independent MCLs that produce a new MCL of size 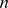. To obtain the number of independent MCLs, one should subtract from 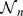 the contributions due to superpositions of smaller pools. For instance, all distinct pairs of pools of size 1 would contribute to 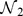 as well, so that the number of independent pools of size 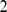 is given by
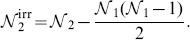
(21)Expression (20) furthermore depends on the particular network being examined. We shall focus on its average over the entire ensemble. Writing the 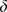-function as 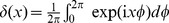, summing over 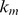
's and averaging over the stoichiometry one finds
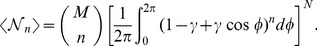
(22)Expanding the integrand and noting that
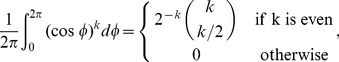
(23)one finally obtains
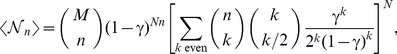
(24)which can be evaluated in the limit 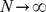. For 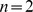, keeping only the leading-order terms in 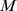 and approximating 
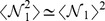
 in (21), one gets
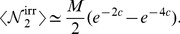
(25)For the networks being examined, once the most connected compounds are removed, one finds 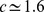, leading to 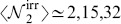 for the *E. coli* core, iJR904 and iAF1260 models, respectively (considering a ‘rich medium’). This should be compared with the actual numbers of independent pools of size 2 we found, namely 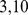 and 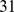 respectively. (Similar results can be obtained, with more work, for larger values of 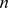.)

It is now straightforward to show that the size 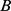 of the pool basis scales linearly with 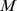. Upon summing (24) over 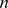, the total number of pools 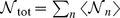 is seen to satisfy
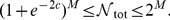
(26)In other terms, there exists a number 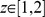 such that 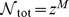. On the other hand, assuming for simplicity that pools in the basis are non-overlapping, one has 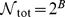, from which we get 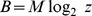, i.e. a linear scaling with 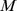, in agreement with the behavior displayed in Figure 1. Therefore, despite the coarse way in which it approximates the considerably more structured real metabolic networks, this ensemble of random networks does provide useful hints about the origin of the scaling behavior observed in cellular networks.

## Discussion

Conservation laws described by the left kernel of the stoichiometric matrix 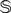 take on a specific biochemical significance when the coefficients involved in their definition are non-negative integers, in which case they typically describe the conservation of a particular molecular moiety. Moiety conservation laws have been shown to have relevant biotechnological or medical implications [51], and are bound to play a key role in understanding the dynamics of intracellular reaction networks. Indeed, conservation relationships, as specific dependencies among variables, allow in principle to reduce the number of degrees of freedom, thereby speeding up dynamical simulations. Unluckily, though, dynamical approaches are still largely prevented from limited knowledge of kinetic constants and reaction mechanisms. Identifying the independent conserved moieties embedded in a given 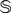, however, requires solving the hard constraint-satisfaction problem of finding all integer, non-negative, solutions to a linear system of equations defined by the network's input-output relationships and corresponding roughly to the maximal conserved moieties introduced in [25]. Methods to tackle this issue on small networks are available and have been employed before. For the network sizes relevant in metabolic modeling, though, the *a priori* search space of the problem is huge and exact methods are doomed to fail because of exceeding computational costs. It is crucial to stress that intractability arises even in presence of a small number of independent MCLs from the fact that retrieving them by a deterministic method would require an exhaustive search through an exponentially large number of candidate solutions. (On the other hand, it is clear that problems with an exponential number of solutions are not necessarily hard to solve.)

Luckily, powerful heuristics to tackle this type of problems has been developed in the last decade at the interface between statistical mechanics and computer science. Here, we have constructed and applied a technique that allows to obtain full information about the independent MCLs associated to a large-scale stoichiometric matrix 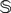 in reasonable compsuting times (a few seconds for genome-scale metabolic networks). In particular, we combine different approaches (message passing, Monte Carlo, and relaxation algorithms) in order to construct a basis for MCLs through which all conserved metabolite pools (i.e. all semi-positive conservation laws) can be described. In short, we first prune irrelevant variables, then extract individual MCLs from the remaining ones, and finally validate our results for completeness of the MCL basis thus obtained. We have analyzed the structure of the MCLs emerging in two large-scale reconstructions of the metabolism of *E. coli* and in the human reactome reconstruction Recon-2, as well as in six tissue-specific subnetworks. In most cases, independent MCLs either display a simple biochemical meaning or their origin can be clearly traced back to properties of the reconstruction. In some cases, however, it is difficult to identify a precise molecular rationale for the groups we obtain. We note however that cofactors like ATP, ADP, AMP, NADH, NADPH, FADH2, etc. do not generically appear in conservation relationships in genome-scale reconstructions. More generally, we have suggested the existence of a linear relation between the number of independent MCLs and the network size, validating it in data and by an analytical calculation for ‘random metabolic networks’, although more work will be needed to characterize this picture more thoroughly.

The foremost advantage of our stochastic method is provided by the limited computing times with respect to the previously defined deterministic methods, which require exponentially large CPU costs in the worst case. It is anyway worth to comment briefly about the appropriateness of the techniques we employ to find independent MCLs. Concerning the Monte Carlo method, the algorithm is a Markov chain verifying the detailed balance condition with respect to the measure (13). Since the states are connected, this condition implies that the chain is ergodic [52]. In very large networks the method may however suffer from effective breakdowns of ergodicity due to exceeding relaxation times. We have however not encountered phenomena of this type on the genome-scale networks we analyzed. On the other hand, the MP protocol we use is formally guaranteed to converge only on tree-like networks, which is a potential drawback. We have however noted that the list of metabolites belonging to at least one MCL can be also computed in other ways, e.g. by analyzing the left kernel of the stoichiometric matrix. This is indeed done explicitly in the C++ code that can be downloaded (together with the test case given by the iAF1260 reconstruction of E. coli's metabolic network) from http://chimera.roma1.infn.it/SYSBIO/, where the overall method we propose is implemented in an automatic way with the only input of a stoichiometric matrix. The fact that MP and kernel analysis provide the same results on each of the 9 networks we tested very strongly suggests that the convergence problems of which MP may suffer on metabolic networks may be less severe than one would have expected. This only confirms the indications obtained from previous MP-based studies of metabolic networks [18]. Unfortunately, MP algorithms are hard to automatize. The possibility to obtain more thorough and potentially useful information through this heuristics (e.g. about the statistics of the vector 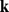 over the solution space) still represents a very high incentive toward their use in our view. For instance, it may be possible to replace the Monte Carlo step by complementing MP with a decimation procedure [52], [53], thus allowing to map out the full set of independent MCLs of a large, genome-scale metabolic network by MP alone, something that would be unfeasible by Monte Carlo.

Besides their importance for dynamical modeling widely discussed in the literature [22], [26], [27], [51], MCLs (or, more generally, the SPCLs that they generate) provide crucial indications concerning how a cell will respond to a perturbation that e.g. increases the level of a particular chemical species. The manner in which that perturbation propagates is indeed constrained by the map of MCLs. In addition, results obtained here can improve producibility predictions [19]. The technique we have presented is successful in large, genome-scale models, and applicability to other organisms is straightforward. More interestingly, however, it may represent a general protocol by which different stoichiometry-based problems that are inherently integer programming ones can be tackled. For instance, a similar approach may form the basis of a method that will tackle the potentially harder problem of computing all extreme pathways of a reaction network at genome-scale resolution [13]. The main difference with the case discussed here is that, while computing the independent MCLs of a genome-scale metabolic network means finding a non-negative integer basis for the SPCLs described by the left kernel of the stoichiometric matrix, extreme pathways form an outright Hilbert basis of the right kernel of the stoichiometric matrix, and the number of elements of a Hilbert basis can be much larger than that of a simple basis. Nevertheless, the existence of useful upper bounds [54] and the excellent performance of the algorithm we introduce may combine to provide cost-effective heuristics for this problem as well. We finally point out that the general issue of producibility in metabolic networks goes beyond purely stoichiometric (mass) constraints and it should include thermodynamics constraints as well. This can be addressed with similar computational techniques, an aspect that we will leave for further studies.

## Supporting Information

Text S1 **Detailed description of the methods, description of the C++ code downloadable (with a text case) from** http://chimera.roma1.infn.it/SYSBIO/ **, and scaling of the number of independent MCLs with the network size in human metabolic reconstructions.**
(PDF)

Material S1 **Independent MCLs found in the human reactome Recon-2 as well as in the six tissue-specific networks tested.**
(GZ) Click here for additional data file.

## References

[1] Jeong H, Tombor B, Albert R, Oltvai Z, Barabasi AL (2000) The large-scale organization of metabolic networks. Nature 407: 651–654. 11034217 10.1038/35036627

[2] Fell D, Wagner A (2000) The small world of metabolism. Nature Biotech 18: 1121–1122. 10.1038/81025 11062388

[3] Dongxiao Z, Zhaohui S (2005) Structural comparison of metabolic networks in selected single cell organisms. BMC Bioinformatics 6: 8. 15649332 10.1186/1471-2105-6-8 PMC549204

[4] Reed J, Vo T, Schilling C, Palsson B (2003) An expanded genome-scale model of escherichia coli k-12 (iJR904 GSM/GPR). Genome Biol 4: R54. 12952533 10.1186/gb-2003-4-9-r54 PMC193654

[5] Palsson B (2006) Systems biology: properties of reconstructed networks. Cambridge (UK): Cambridge University Press.

[6] Almaas E, Kovacs B, Vicsek T, Oltval Z, Barabasi AL (2004) Global organization of metabolic uxes in the bacterium escherichia col. Nature 427: 839–843. 14985762 10.1038/nature02289

[7] Segré D, Vitkup D, Church G (2002) Analysis of optimality in natural and perturbed metabolic networks. Proc Nat Acad Sci USA 99: 15112–15117. 12415116 10.1073/pnas.232349399 PMC137552

[8] Joyce A, Palsson B (2008) Predicting gene essentiality using genome-scale in silico models. Methods Mol Biol 416: 433–457. 18392986 10.1007/978-1-59745-321-9_30

[9] Schellenberger J, Que R, Fleming R, Thiele I, Orth J, et al (2011) Quantitative prediction of cellular metabolism with constraint-based models: the cobra toolbox v2.0. Nature Protocols 6: 1290–1307. 21886097 10.1038/nprot.2011.308 PMC3319681

[10] Heinrich R, Schuster S (1996) The Regulation of Cellular Systems. Berlin: Springer.

[11] Beard D, Qian H (2008) Chemical biophysics. Cambridge (UK): Cambridge University Press.

[12] Behre J, de Figueiredo L, Schuster S, Kaleta C (2012) Detecting structural invariants in biological reaction networks. In: Bacterial Molecular Networks: Methods and Protocols (Methods in Molecular Biology, vol. 804). pp. 377–407. 10.1007/978-1-61779-361-5_20 22144164

[13] Yeung M, Thiele I, Palsson B (2007) Estimation of the number of extreme pathways for metabolic networks. BMC Bioinformatics 8: 363. 17897474 10.1186/1471-2105-8-363 PMC2089122

[14] Papin J, Price N, Edwards J, Palsson B (2002) The genome-scale metabolic extreme pathway structure in haemophilus inuenzae shows significant network redundancy. J Theor Biol 215: 67–82. 12051985 10.1006/jtbi.2001.2499

[15] Schuster S, Dandekar T, Fell D (1999) Detection of elementary ux modes in biochemical networks: a promising tool for pathway analysis and metabolic engineering. Trends Biotechnol 17: 53–60. 10087604 10.1016/s0167-7799(98)01290-6

[16] Wiback S, Famili I, Greenberg H, Palsson B (2004) Monte carlo sampling can be used to determine the size and shape of the steady-state ux space. J Theor Biol 228: 437–447. 15178193 10.1016/j.jtbi.2004.02.006

[17] Price N, Schellenberger J, Palsson B (2004) Uniform sampling of steady-state ux spaces: means to design experiments and to interpret enzymopathies. Biophys J 87: 2172–2186. 15454420 10.1529/biophysj.104.043000 PMC1304643

[18] Braunstein A, Mulet R, Pagnani A (2008) Estimating the size of the solution space of metabolic networks. BMC Bioinformatics 9: 240. 18489757 10.1186/1471-2105-9-240 PMC2483728

[19] Martelli C, De Martino A, Marinari E, Marsili M, Perez Castillo I (2009) Identifying essential genes in escherichia coli from a metabolic optimization principle. Proc Nat Acad Sci USA 106: 2607–2611. 19196991 10.1073/pnas.0813229106 PMC2636734

[20] Thiele I, Price N, Vo T, Palsson B (2005) Candidate metabolic network states in human mitochondria. impact of diabetes, ischemia, and diet. J Biol Chem 280: 11683–11695. 15572364 10.1074/jbc.M409072200

[21] Schuster S, Höfer T (1991) Determining all extreme semi-positive conservation relations in chemical reaction systems: a test criterion for conservativity. J Chem Soc Faraday Transac 87: 2561–2566.

[22] Famili I, Palsson B (2003) The convex basis of the left null space of the stoichiometric matrix leads to the definition of metabolically meaningful pools. Biophys J 85: 16–26. 12829460 10.1016/S0006-3495(03)74450-6 PMC1303061

[23] Papadimitrou C, Steiglitz K (2000) Combinatorial optimization: algorithms and complexity. Dover Publications Inc.

[24] Conti P, Traverso C (1991) Buchberger algorithm and integer programming. In: Lectures Notes in Computer Science Vol. 539. Berlin: Springer, pp. 130–139.

[25] Schuster S, Hilgetag C (1995) What information about the conserved-moiety structure of chemical reaction systems can be derived from their stoichiometry? J Phys Chem 99: 8017–8023.

[26] Vallabhajosyula R, Chickarmane V, Sauro H (2006) Conservation analysis of large biochemical networks. Bioinformatics 22: 346–353. 16317075 10.1093/bioinformatics/bti800

[27] Sauro H, Ingalls B (2004) Conservation analysis in biochemical networks: computational issues for software writers. Biophys Chem 109: 1–15. 15059656 10.1016/j.bpc.2003.08.009

[28] Nikolaev E, Burgard A, Maranas C (2005) Elucidation and structural analysis of conserved pools for genome-scale metabolic reconstructions. Biophys J 88: 37–49. 15489308 10.1529/biophysj.104.043489 PMC1305013

[29] Imielinski M, Belta C, Rubin H, Halász A (2006) Systematic analysis of conservation relations in escherichia coli genome-scale metabolic network reveals novel growth media. Biophys J 90: 2659–2672. 16461408 10.1529/biophysj.105.069278 PMC1414550

[30] Gevorgyan A, Poolman M, Fell D (2008) Detection of stoichiometric inconsistencies in bimolecular models. Bioinformatics 24: 2245–2251. 18697772 10.1093/bioinformatics/btn425

[31] Koch I (2010) Petri nets: A mathematical formalism to analyze chemical reaction networks. Molecular Informatics 29: 838–843. 27464348 10.1002/minf.201000086

[32] Thiele I, Swainston N, Fleming RMT, Hoppe A, Sahoo S, et al (2013) A community-driven global reconstruction of human metabolism. Nature Biotechnol 31: 419–425. 23455439 10.1038/nbt.2488 PMC3856361

[33] Henk M, Weismantel R (1997) On Hilbert bases of polyhedral cones. Results Math 32: 298–303.

[34] Coleman T, Pothen A (1986) The null space problem I. Complexity. SIAM J Alg Discr Meth 7: 527–537.

[35] Contejean E, Devie H (1994) An efficient incremental algorithm for solving systems of linear Diophantine equations. Inform & Comput 113: 143–172.

[36] Pasechnik V (2001) On computing the Hilbert bases via the Elliott-MacMahon algorithm. Theor Comp Sci 263: 37–46.

[37] Braunstein A, Mezard M, Zecchina R (2005) Survey propagation: an algorithm for satisfiability. Random Struct Algor 27: 201–226.

[38] Baldassi C, Braunstein A, Brunel N, Zecchina R (2007) Efficient supervised learning in networks with binary synapses. Proc Nat Acad Sci USA 104: 11079–11084. 17581884 10.1073/pnas.0700324104 PMC1904114

[39] Mulet R, Pagnani A, Weigt M, Zecchina R (2002) Coloring random graphs. Phys Rev Lett 89: 268701. 12484862 10.1103/PhysRevLett.89.268701

[40] Pearl J (1982) Reverend bayes on inference engines: A distributed hierarchical approach. In: Proc. AAAI Nat. Conf. AI (Pittsburgh, PA). pp. 133–136.

[41] Kim J, Pearl J (1983) A computational model for causal and diagnostic reasoning in inference systems. In: Proceedings IJCAI-83 (Karlsruhe, Germany). pp. 190–193.

[42] Mezard M, Montanari A (2009) Information, physics, and computation. Oxford University Press.

[43] Yedidia JS, Freeman WT, Weiss Y (2000) Generalized belief propagation. In: Neural Information Processing Systems 13. MIT Press, pp. 689–695.

[44] Kschischang F, Frey B, Loeliger H (2001) Factor graphs and the sum-product algorithm. IEEE Transactions on Information Theory 47: 498–519.

[45] Krauth W (1998) Introduction to monte carlo algorithms. Advances in Computer Simulations 501: 1–35.

[46] Imielinski M, Belta C, Rubin H, Halász A (2005) Investigating metabolite essentiality through genome-scale analysis of escherichia coli production capabilities. Bioinformatics 12: 2008–2016. 10.1093/bioinformatics/bti245 15671116

[47] De Martino A, Martelli C, Monasson R, Perez Castillo I (2007) Von Neumann's expanding model on random graphs. JSTAT 2007: P05012.

[48] Schrijver A (1986) Theory of linear and integer programming. Chichester: Wiley.

[49] Krauth W, Mezard M (1987) Learning algorithms with optimal stability in neural networks. J Phys A: Math Gen 20: L745–L752.

[50] Feist A, Henry C, Reed J, Krummenacker M, Joyce A, et al (2007) A genome-scale metabolic reconstruction for escherichia coli K-12 MG1655 that accounts for 1260 ORFs and thermodynamic information. Mol Sys Biol 3: 121. 10.1038/msb4100155 PMC1911197 17593909

[51] Bakker B, Michelsi P, Opperdoesi F, Westerhoff H (1999) What controls glycolysis in bloodstream form trypanosoma brucei? J Biol Chem 274: 14551–14559. 10329645 10.1074/jbc.274.21.14551

[52] Mezard M, Parisi G, Zecchina R (2002) Analytic and algorithmic solution of random satisfiability problems. Science 297: 812–815. 12089451 10.1126/science.1073287

[53] Ricci Tersenghi F, Semerjian G (2009) On the cavity method for decimated random constraint satisfaction problems and the analysis of belief propagation guided decimation algorithms. JSTAT 2009: P09001.

[54] Eisenbrand F, Shmonin G (2006) Caratheodory bounds for integer cones. Operations Research Letters 34: 564–568.

