# Supplementary material for: Identifying All Moiety Conservation Laws in Genome-Scale Metabolic Networks
Source: PLoS One. 2014 Jul 2;9(7):e100750. doi: 10.1371/journal.pone.0100750 (PMC4079565; doi:10.1371/journal.pone.0100750)
Supplement: Text S1 — Detailed description of the methods, description of the C++ code downloadable (with a text case) from http://chimera.roma1.infn.it/SYSBIO/ , and scaling of the number of independent MCLs with the network size in human metabolic reconstructions. (PDF) [file pone.0100750.s001.pdf]

Identifying all moiety conservation laws in genome-scale metabolic networks – SUPPORTING TEXT

Andrea De Martino[1,2,3,⋆], Daniele De Martino[3,⋆], Roberto Mulet[4,⋆], Andrea Pagnani[5,6,⋆]

1 CNR-IPCF, Unità di Roma-Sapienza, Roma (Italy)
2 Dipartimento di Fisica, Sapienza Università di Roma, p.le A. Moro 2, 00185 Roma, Italy
3 Center for Life Nano Science@Sapienza, Istituto Italiano di Tecnologia, Viale Regina Elena 291, 00161 Roma (Italy)
4 Henri-Poincaré-Group of Complex Systems and Department of Theoretical Physics, Physics Faculty, University of Havana, CP 10400 La Habana (Cuba)
5 DISAT and Centre for Computational Sciences, Politecnico di Torino, Corso Duca degli Abruzzi 24, 10129 Torino (Italy)
6 Human Genetics Foundation, Via Nizza 52, 10126 Torino (Italy)

⋆ *All authors contributed equally to this work*

Contents

1 Methods: further details **2**
1.1 Message-Passing . . . 2
1.2 Monte Carlo . . . 3
1.3 Relaxation . . . 4

2 Overall implementation: description of the code **4**

3 Scaling of the size of the MCL basis: human networks **6**

1 Methods: further details

1.1 Message-Passing

We aim at computing the marginals

$$P_m(k_m) \equiv \sum_{\{k_n\}_{n\neq m}} P(\mathbf{k}) \tag{1}$$

of the probability distribution

$$P(\mathbf{k}) = \frac{1}{\mathcal{N}_{\rm sol}} \prod_{i=1}^{N} \delta\left(\sum_{m=1}^{M} S_{m,i} k_m ; 0\right) \quad , \quad k_m \in \{0,1,2,\ldots\} \ \forall m \quad , \tag{2}$$

where $\delta(x;y)$ is the Kronecker delta function ($=1$ if $x=y$ and $=0$ otherwise) and $\mathcal{N}_{\rm sol}$ stands for the number of solutions of the system of equations

$$\mathbb{S}^T \mathbf{k} = \mathbf{0} \qquad , \qquad \mathbf{k} \neq \mathbf{0} \quad , \quad k_m \in \{0,1,2,\ldots\} \ \forall m \quad . \tag{3}$$

Let us define the cost function

$$H(\mathbf{k}) = \sum_{i=1}^{N} \left[1 - \delta\left(\sum_{m=1}^{M} S_{m,i} k_m ; 0\right)\right] \quad , \quad k_m \in \{0,1,2,\ldots\} \ \forall m \quad . \tag{4}$$

Clearly, $H \geq 0$. In addition, $H = 0$ when $\mathbf{k}$ is a solution of (3), i.e. when it defines a MCL. In essence, the message-passing (MP) algorithm we employed is based on nodes (metabolites and reactions) exchanging two types of 'messages':

- $\mu_{i\to m}(k)$ (from reactions to metabolites), representing the (non-normalized) probability that the constraint imposed by the i-th equation in (3), i.e. $\sum_n S_{n,i} k_n = 0$, is fulfilled given that the m-th variable takes on value k;
- $\rho_{m\to i}(k)$ (from metabolites to reactions), giving the probability that the m-th variable takes on value k in the absence of reaction i.

On a tree-like network, these quantities can be shown to be related by [1]

$$\mu_{i\to m}(k_m) = \sum_{\{k_n\}_{n\neq m}} \delta\left(\sum_{n=1}^{M} S_{n,i} k_n ; 0\right) \prod_{n\in i\setminus m} \rho_{n\to i}(k_n) \tag{5}$$

$$\rho_{m\to i}(k_m) = C_{m\to i} \prod_{j\in m\setminus i} m_{j\to m}(k_m) \tag{6}$$

where $C_{m\to i}$ is a constant enforcing the normalization of the probability $\rho_{m\to i}(k_m)$, and the subscript $j \in m\setminus i$ denotes the set of reactions producing or consuming metabolite m but reaction i (and similarly for the subscript $n \in i\setminus m$). The above equations can be solved iteratively by initializing messages at random and updating them in a random sequential order. The algorithm halts when the difference between each message at iteration t and iteration $t-1$ is less than a pre-defined threshold. Once convergence is reached, the desired marginal probability distribution can be computed as the product of all μ-messages pointing to the m-th variable, namely

$$P_m(k_m) = C_m \prod_{j\in m} \mu_{j\to m}(k_m) \quad , \tag{7}$$

where C_m is, again, a normalization constant.

Although rigorously exact only on tree-like structures, Equations (5), (6) and (7) have been extensively employed for the analysis of loopy networks (including metabolic networks [2]), the key correctness test being ultimately their ability to find solutions. In a nutshell, the approximation is sufficiently good when the corrections induced by the presence of loops are negligible, as appears to be the case for the problem at stake. We therefore applied the above MP scheme (known as Belief Propagation, BP) to (3). As a threshold for convergence, we chose 10^{-8} in all our simulations. While the number of iterations is generically problem-dependent, in the simulations presented here it rarely exceeds a few hundreds. Moreover, in order to account for possible dependencies of results on the initialization of messages, we have repeated the procedure for 20 different choices of the initial conditions, and used, as the final list of metabolites belonging to at least one MCL, the union of the different outputs. The lists obtained by different initial conditions however varied at most by few metabolites (3 or less) among each other.

To conclude, we note that, by using BP, it is also possible to compute conditional marginal probability distributions, i.e. the marginal probability distribution that the m-th variable takes value k given that a set of ℓ other variables $\{n_1, \ldots, n_\ell\}$ take on values $\hat{k}_{n_1}, \ldots, \hat{k}_{n_\ell}$ respectively. This can be done, in specific, by adding an appropriate set of 'external fields' to the cost function. Note indeed that

$$P(\mathbf{k}) = \lim_{T\to 0} P_T(\mathbf{k}) \qquad , \qquad P_T(\mathbf{k}) = \frac{e^{-H(\mathbf{k})/T}}{Z(T)} \tag{8}$$

where $T \geq 0$ is a parameter and $Z(T)$ is a normalization factor (the limit $T \to 0$ concentrates $P(\mathbf{k})$ around the minima of H) and consider the transformation

$$H(\mathbf{k}) \to H(\mathbf{k}) + T \sum_{n=1}^{M} h_n \delta(k_n; \hat{k}_n) \quad , \tag{9}$$

By setting

$$h_n = \begin{cases} h_0 \gg 1 & \text{for } n \in \{n_1, \ldots, n_\ell\} \\ 0 & \text{otherwise} \end{cases} \tag{10}$$

one may set variables $k_{n_1}, \ldots, k_{n_n}$ to the desired values $\hat{k}_{n_1}, \ldots, \hat{k}_{n_\ell}$ and thus force the BP iteration to converge to the correct conditional probability distribution as $T \to 0$. This can be especially useful if one is interested in extracting solutions where k_m attains a precise value (i.e. where metabolite m appears in a specific way).

1.2 Monte Carlo

For our study, we employed a simple Metropolis scheme defined as follows: at each step, select a variable k_m at random among those appearing in the list of metabolites belonging to at least one MCL obtained by BP, and propose an update of the form

$$k_m \to k_m + \delta \quad , \tag{11}$$

where $\delta = \pm 1$ with equal probability if $k_m > 0$ and $\delta = 1$ if $k_m = 0$. Next, evaluate the ensuing change of the 'energy' E given by (see main text)

$$E(\mathbf{k}) = \sum_{m,n=1}^{M_c} J_{m,n} k_m k_n \geq 0 \qquad , \qquad \text{with} \quad J_{m,n} \equiv \sum_{i=1}^{N} S_{m,i} S_{n,i} \quad , \tag{12}$$

namely

$$\Delta E = 2\delta \sum_{n \neq m} J_{m,n} k_n + J_{m,m} \quad . \tag{13}$$

(we recall that M_c denotes the number of metabolites belonging to at least one MCL). The proposed move is then accepted with probability

$$P(\text{accept}) = \arg\ \min\ \{1, e^{-\Delta E/T}\}\ , \tag{14}$$

where $T \geq 0$ is a parameter. For each choice of T, this Markov chain converges to the probability distribution [3]

$$P(\mathbf{k}) = \frac{e^{-E(\mathbf{k})/T}}{Z(T)}\ , \tag{15}$$

with a mixing time that increases as T gets smaller and depends on the distribution of initial states (so that the closer the latter is to (15), the smaller the mixing time) [4]. (Note that for $T = 0$ the above dynamics becomes a gradient descent.) The minima of E are recovered in the limit $T \to 0$, which can be achieved operationally by initializing the Monte Carlo simulation at some large value of T and then decreasing the 'temperature' T at a constant rate until $T = 0$, a procedure known as 'annealing'. When decresing T with a fixed schedule, it is possible to speed up the convergence by using, as the initial state for a certain T, the final state at the previous (higher) value of T.

1.3 Relaxation

As explained in the main text, the relaxation step of our protocol converges (in polynomial time) to a solution of the dual problem with a reduced stoichiometric matrix (i.e., $\mathbb{S}$ without the rows corresponding to metabolites belonging to at least one MCL) if all MCLs have been found, and this property constitutes a certificate for completeness of the MCL basis retrieved by the combination of MP (or kernel analysis) and Monte Carlo.

If no solution is found, however, the reduced $\mathbb{S}$ necessarily harbors more pools, which MP was possibly unable to locate (we recall that MP is only approximate for loopy networks). In order to identify the missing MCLs in the reduced $\mathbb{S}$, one may proceed by analyzing the dynamics of the relaxation algorithm similarly to what was done for the mathematically related problem of identifying thermodynamically infeasible cycles in flux patterns [5]. As discussed in detail e.g. in [5,6], after a transient, the relaxation algorithm visits frequently the constraints that prevent convergence. Therefore, MCLs can be easily identified by keeping tracks of the least unsatised metabolite constraints (denoted by $\underline{m}(t)$ in the main text) over time.

2 Overall implementation: description of the code

A C++ code that performs our method, returning a list of independent MCLs using a stoichiometric matrix $\mathbb{S}$ as the sole input, is provided as Supporting Material and can be downloadad from http://chimera.roma1.infn.it/SYSB Because MP is generically harder to automatize than Monte Carlo and relaxation methods, we have opted to replace it, in the supporting code, with an elementary pre-processing step in which the left kernel of $\mathbb{S}$ is analyzed (see main text). In essence, the code begins by computing the properties of the left kernel of $\mathbb{S}$ to derive the list of metabolites belonging to at least one MCLs. While MP gives in principle much more information (contained in the marginals $P_m(k_m)$ defined above), for the simple sakes of extracting independent MCLs a list of candidate metabolites obtained by a simpler method suffices. In summary, then, the supplied procedure includes (a) preprocesing by analysis of Ker($\mathbb{S}^T$); (b) simulated annealing Monte Carlo to extract irreducible MCLs; (c) Motzkin-type relaxation to check for completeness.

The algorithm diagram is sketched in Fig. 1. In brief, the algorithm runs as follows. First, Ker($\mathbb{S}^T$) is calculated through Gaussian elimination with scaled pivoting. The dimension of the kernel is retained together with all the vectors in the kernel basis that have a unique (positive) sign of the components (i.e. all SPCLs, see main text). If all the vectors of the kernel have the form of MCLs (i.e. if the coefficients

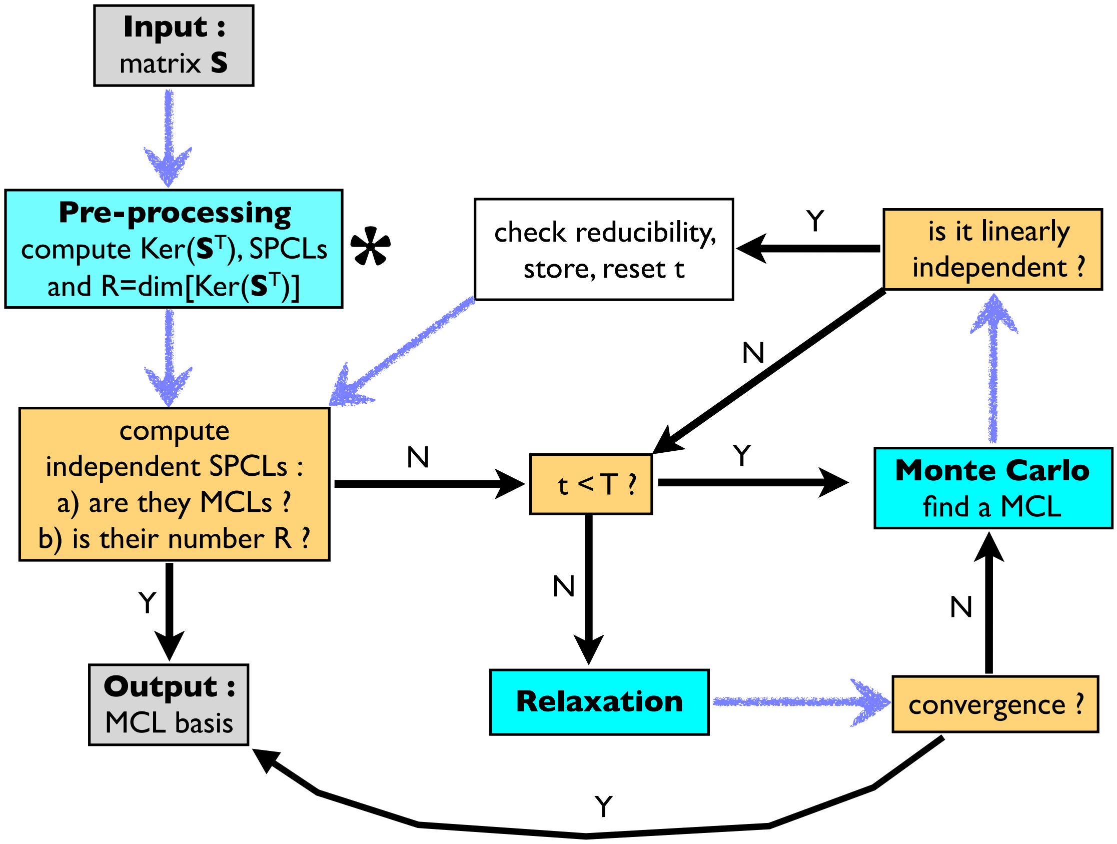

Figure 1. Flow chart of the overall method as performed by the C++ code provided as Supporting Material and downloadable from http://chimera.roma1.infn.it/SYSBIO/. After the pre-processing step in which SPCLs are computed, the code checks whether independent SPCLs are in fact MCLs. If yes, it halts, returning the MCL basis. If not, it enters the Monte Carlo routine (provided this has not yet happened T times, with T a sufficiently large number representing the maximum number of allowed Monte Carlo runs). If the MCL found by Monte Carlo is linearly from those previously found, a reducibility check is performed and the solution is stored. If not, a new solution is searched for, unless the Monte Carlo step has been performed the maximum allowed number of times. In this case, a completeness check is started by running Motzkin relaxation as explained in the main text. In more advanced, though less automatizable versions of the protocol, the pre-processing step (indicated by the asterisk) is replaced by the message-passing scheme.

are integers) and if their number matches the dimension of the kernel, the output is precisely the MCL basis we are looking for. If not (which is normally the case), the parameters $J_{m,n}$ in (12) are computed for the metabolites appearing with non-zero components in the vectors of the kernel and a Monte Carlo simulated annealing is performed with energy E given in (12). After searching for and removing linear dependencies, a relaxation run is launched to verify completeness.

3 Scaling of the size of the MCL basis: human networks

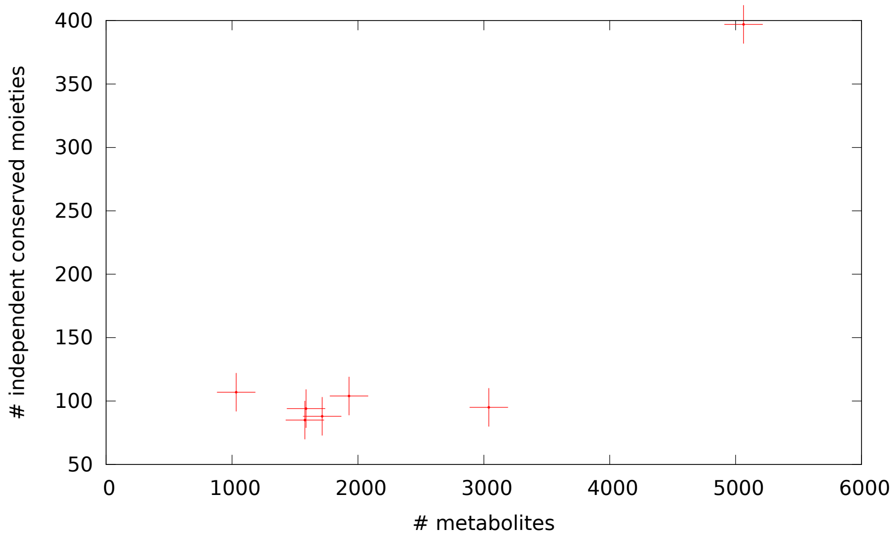

Figure 2. Size of the MCL basis as a function of the number of metabolites (M) for six tissue-specific reconstructions of human metabolic networks (see Table 5, main text) and for the Recon-2 reactome ($M = 5063$).

References

1. Mezard M, Montanari A (2009) Information, physics, and computation. Oxford University Press.

2. Braunstein A, Mulet R, Pagnani A (2008) Estimating the size of the solution space of metabolic networks. BMC Bioinformatics 9: 240.

3. Krauth W (1998) Introduction to monte carlo algorithms. Advances in Computer Simulations 501: 1-35.

4. Binder K, Heermann D (2010) Montecarlo simulation in statistical physics: an introduction. Heidelberg: Springer.

5. De Martino D, Figliuzzi M, De Martino A, Marinari E (2012) A scalable algorithm to explore the gibbs energy landscape of genome-scale metabolic networks. PLoS Comp Biol 8: e1002562.

6. De Martino D (2013) Thermodynamics of biochemical networks and duality theorems. Phys Rev E 87: 052108.
